# Echocardiography Monitoring during Anthracycline Administration in Hodgkin and Non-Hodgkin’s Lymphoma: The Tei Index Evaluation

**DOI:** 10.3390/jpm12020290

**Published:** 2022-02-16

**Authors:** Agata Puzzovivo, Agnese Maria Fioretti, Carla Minoia, Roberta Villoni, Santa Carbonara, Giusi Graziano, Fabio Pavone, Attilio Guarini, Stefano Oliva

**Affiliations:** 1 Cardioncology Unit, IRCCS IstitutoTumori “Giovanni Paolo II”, 70124 Bari, Italy; amfioretti@libero.it (A.M.F.); r.villoni@oncologico.bari.it (R.V.); stefanoliva66@gmail.com (S.O.); 2 Hematology Unit, IRCCS IstitutoTumori “Giovanni Paolo II”, 70124 Bari, Italy; c.minoia@oncologico.bari.it (C.M.); fabio.pavone.irccsbari@gmail.com (F.P.); attilioguarini@oncologico.bari.it (A.G.); 3 Section of Cardiovascular Diseases, Department of Emergency and Organ Transplantation (DETO), University Policlinic Hospital, 70124 Bari, Italy; titticarbo@hotmail.it; 4 Scientific Direction, IRCCS IstitutoTumori “Giovanni Paolo II”, 70124 Bari, Italy; giusi.graziano78@gmail.com

**Keywords:** lymphoma, Tei index, echocardiography, cardiotoxicity, personalized medicine

## Abstract

Anthracyclines are widely employed in lymphoma’s chemotherapy and has been shown to induce heart failure. Echocardiographic parameters of left ventricular (LV) systolic function are usually used to monitor the cardiac side effects during and after anthracyclines treatment. The measurement of theTei index could anticipate the onset of LV dysfunction. The aim of this study was to evaluate the performance of the delta Tei index for the early detection of cardiac toxicity in a prospective population of anthracycline-treated lymphoma patients. Our preliminary data suggest that the Tei index may predict the risk for cardiotoxicity in this subset of patients earlier than LV ejection fraction alteration.

## 1. Introduction

Anthracycline therapy continues to be the cornerstone in the treatment of Hodgkin (HL) and non-Hodgkin’s lymphoma (NHL), with the best known limitation being the potential dose-dependent early and late cardiotoxicity [1,2]. Different cardiotoxic effects can occur during and after anthracycline administration within a poli-chemotherapy schedule, but the most feared is left ventricular systolic damage, ranging from asymptomatic left ventricular systolic dysfunction (LVSD), recognized as a reduction of left ventricular ejection function (LVEF) below the limit of normality, to congestive heart failure (CHF). LVSD can manifest within one year of completing chemotherapy (early cardiotoxicity) or after many years (late cardiotoxicity) [2,3]. In fact, anthracyclines determineirreversible microscopic alterations resulting in increased myocardiocytic apoptosis, myocardial edema and cardiac fibrosis [1,2]. Macroscopically, anthracycline-related cardiotoxicity causes reduction of left ventricular (LV) mass, thinning of cardiac walls and LV dilatation [1]. This “cardiotoxic phenomenon” appears to be mediated by an increased intracellular calcium concentration and a dysregulation of the myocardial survival/regeneration pathway, crucial mechanisms for maintaining LV systo-diastolic function [2].

The cumulative dose (more than 250 mg/m^2^ for doxorubicin, 300 mg/m^2^ for epirubicine), patient’s age, family history of CHF and the clinical risk factors (hypertension, diabetes, hypercholesterolemia, left ventricular hypertrophy, etc.) seem to be related to the higher risk of developing signs and symptoms of heart failure. Currently, there is no score available to predict the individual risk of anthracycline-related toxicity, but a scheduled follow-up according to multiple risk factors has been recently proposed [4,5]).

The position paper of the European Society of Cardiology (ESC) [6] suggests the monitoring of LVEF as the main preventive strategy in cancer patients, even if the monitoring of cardiac biomarkers (troponin I) is also recommended, as demonstrated by issues affecting patients during a high-dose chemotherapy regimen [7]. The ESC paper proposes a definition of chemotherapy-related cardiotoxicity as a LVEF decrease of >10 percentage points from baseline to a value <53% on repeating confirmatory echocardiographic imaging [6]. The reduction of LVEF is a powerful predictor of clinical outcomes and mortality, as has been widely documented in literature. However, LVEF is not a highly sensitive tool because it identifies patients with significant and often irreversible alterations of contractility, focusing just on systolic function. Moreover, LVEF is characterized by an inter-observer variability, with reliance on good two-dimensional images and geometric assumptions [8,9]. For this reason, it is essential to assess an echocardiographic marker of early LVdysfunction capable of precociously identifying patients at greater risk of developing cardiotoxicity. The global longitudinal strain (GLS) value seems to decrease in cancer patients, earlier than LVEF [10]. A relative percentage reduction of GLS of >15% from baseline is considered abnormal and a marker of early LV subclinical dysfunction. However, the ESC task force declares that these suggested strategies have not been validated, and so, at present, there is not even one shared and sure preventive strategy for CHF [6]. Other authors affirmed that in cancer patients the antiblastic therapy with anthracyclines modifies the left ventricular diastolic function (LVDF) before the systolic function [8]. There are many reasons that point to not using LVDF monitoring in routine practice, primarily its low sensitivity as regards the ability to identify the real dysfunctional patients.

Other systolic and diastolic echocardiographic parameters have been studied over the last years, but none havedemonstrated correlation to cardiotoxic events during follow-up in large randomized trials. The Tei index is a parameter of systo-diastolic function that can be calculated during a 2D transthoracic echocardiography (TTE) using a doppler wave of mitral and aortic valve, as sum of the isovolumetric relaxation and contraction time divided by the ejection time [11]. One trial showed that the Tei index change (ΔTei index) significantly correlates with an increasing dose of doxorubicin, also in hematologic patients treated with anthracyclines. However, the increasing dose of doxorubicin was not related to the LVEF and, furthermore, the ΔTei index did not depend on either the Tei index value or LVEF value [12]. Zhang CJ and colleagues demonstrated that the echocardiographic PW Doppler-derived Tei index, and TDI-derived Sm, combined with serum hs-cTnT level, could be obtained in outpatient settings to monitor early cardiac toxicity induced by anthracycline therapy [13]. There are no other trials that confirm these results in a higher number of patients and, above all, that show a correlation between increasing Tei index and cardiotoxicity.

The aims of this prospective study were (I) to demonstrate that a modification of the Tei index (ΔTei index) during serial echocardiographic exams is a useful parameter for the detection of early cardiotoxicity in hematologic patients receiving an anthracycline-containing regimen as up-front therapy for HL or NHL, and (II) to assess whether this parameter had a higher and earlier predictive value with respect to LVEF or the appearance of signs and symptoms of CHF.

## 2. Materials and Methods

We prospectively evaluated consecutive outpatients affected by HL and NHL treated with ABVD (doxorubicin, bleomycin, vinblastine, dacarbazine) or R-CHOP (rituximab, cyclophosphamide, doxorubicin, vincristine, prednisolone), respectively, and referred to the Cardiology Unit of IRCCS Istituto Tumori “Giovanni Paolo II” in Bari. Inclusion criteria were: age ≥18 years old, at least four courses of anthracycline-containing regimen planned (≥200 mg/m^2^), and signed informed consent. Exclusion criteria were: arrhythmias (atrial fibrillation or atrial flutter), cardiomyopathies, moderate or severe left-ventricular hypertrophy, and a low LVEF (LVEF ≤ 45%) value measured before the start of therapy.

We performed a two-dimensional TTE at precise time points: (1) before the first course of chemotherapy, (2) at different chemotherapy courses, (3) at the end of the planned chemotherapy, and (4) at one year from the end of chemotherapy. The exams were carried out according to standard guidelines [8]. Blood samples were obtained from all subjects.

The Research Ethics Committee of our Institute reviewed and approved the study protocol, which conforms to the ethical guidelines of the Declaration of Helsinki on the principles for medical research involving human subjects [14]. Informed consent was obtained from each patient.

### 2.1. Baseline Evaluations

At the time of enrolment, all the patients underwent a medical visit, an ECG, a two-dimensional TTE, and a blood sample for chemical evaluations.

### 2.2. Medical Examination and Electrocardiogram

A documented record was made for each patient, including ischemic heart disease, arterial hypertension and diabetes mellitus diagnosis, as well as a history of arrhythmic events. During the medical examination, arterial pressure was evaluated, whereas heart rhythm and rate were assessed by 12-lead ECG.

### 2.3. Echocardiographic Evaluation and Tei Index Measurement

All the patients underwent an echocardiographic evaluation using an echocardiograph (Hewlett Packard SONOS 2500). A two-dimensional parasternal long-axis view of the left ventricle was obtained and the following parameters were evaluated: aortic root diameter at the level of Valsalva sinuses (expressed in millimeters [mm]), left ventricular end-diastolic diameter (LVEDD) (mm), left ventricular end-systolic diameter (LVESD) (mm). In the apical four chamber view, we calculated the LV end-diastolic volume (EDV), end-systolic volume (ESV) and stroke volume (SV) by the conventional method. LVEF (left ventricular ejection fraction): ejection fraction is the volume ejected from the left ventricle as a percentage of end-diastolic volume. LVEF is calculated from EDV and ESV estimates, using the following formula: (EDV-ESV)/EDV, with the biplane Simpson method. The biplane method of disks (a modified derivative of Simpson’s rule) is the currently-recommended 2D method to assess LVEF by consensus of the American Society of Echocardiography and the European Association of Cardiovascular Imaging [6]. Pulsed-wave (PW) Doppler was performed in the apical four-chamber view to obtain mitral and tricuspid inflow velocities so as to assess ventricular filling: peak early filling velocity (E), late diastolic filling velocity (A) and the E/A ratio [15].

The Tei index was calculated for both ventricles by the formula (IVRT+IVCT)/ET [11]. All diastolic and time interval parameters were measured in three consecutive cardiac cycles and averaged. Based on the median value of Δ-Tei index at the second cycle of chemotherapy, the patients were divided into two categories with low (Tei-) and with high (Tei+) Tei index change, respectively.

### 2.4. Statistical Analysis

The comparisons of the patients’ baseline characteristics between Tei+ and Tei- group were calculated with the Mann–Whitney U test for continuous variables and the Pearson χ2 test for categorical variables. The variation of other cardiac parameters with time was assessed with the Wilcoxon signed-rank test, separately for the two groups. The correlation between some measurements of interest was calculated with the Pearson’s correlation coefficient (r). The statistical significance was achieved at a *p*-value < 0.05. All the analyses were performed using the Statistical Analysis System (SAS, version 9.4).

## 3. Results

A total of 41 patients were enrolled in the study. Two patients died before the completion of chemotherapy. Two patients were lost at follow-up. Baseline clinical characteristics are shown in Table 1.

Doxorubicin was the most commonly used molecule (93% of patients). There were no significant associations between baseline clinical characteristics and the Tei index value (Table 2).

All studied patients had no significant drop of LVEF in either arm (Tei+ and Tei-), during chemotherapy administration and follow-up. No correlation was demonstrated between the Tei index basal value and LVEF values measured during chemotherapy and follow-up (Table 3). The stroke volume (SV) didnot change either. No patients developed signs or symptoms of CHF. In Table 4, we summarized the LVEF and SV changes between the first and forth cycle of chemotherapy and between the first cycle of chemotherapy and twelvemonths of follow-up.

In the Tei+ patients, the end diastolic volume (EDV) and the end systolic volume (ESV) did not change either during the administration of therapy or during the follow-up On the other hand, in the Tei- patients, we observed a significant increase of EDV and ESV (*p* = 0.04 and *p* = 0.02, respectively) between the first and the fourth cycles (Supplementary Materials, Table S1).

**Figure 1:**
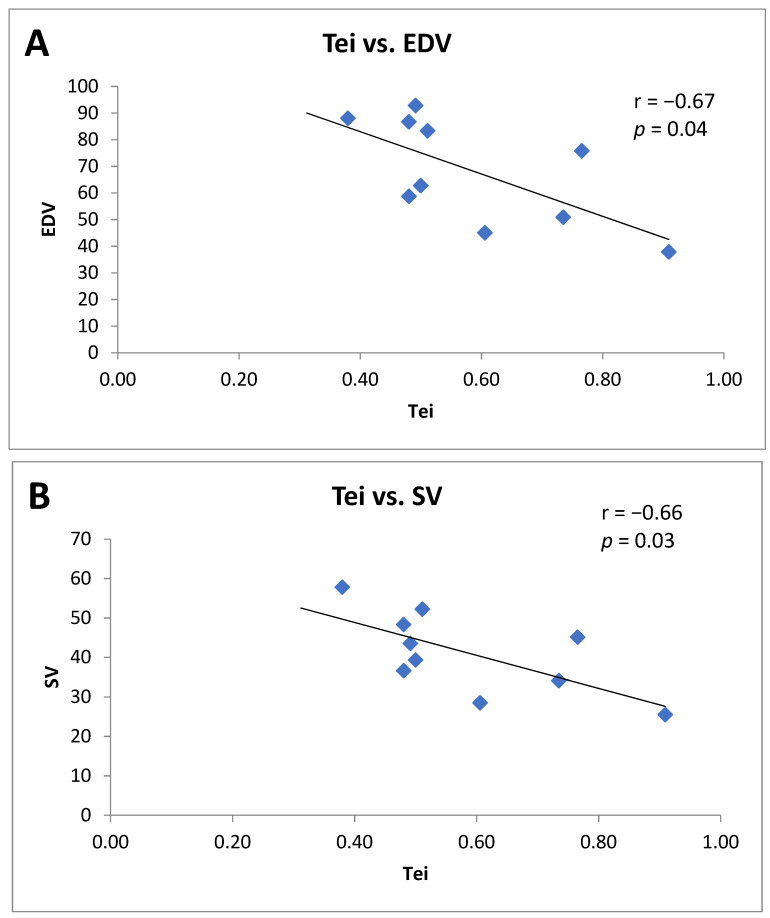
**(A**) Correlation between theTei index value and the end diastolic volume (EDV) at 12 months of follow-up in Tei- patients. (**B**) Correlation between Tei index value and stroke volume (SV) at 12 months of follow-up in Tei- patients.

Furthermore, only in Tei- patients did a significant inverse correlation between the baseline Tei index value and the EDV value (r = −0.67; *p* = 0.04) and the SV value occur(r = −0.66; *p* = 0.03), both measured at 12 months of follow-up (Figure 1, panel A and B).

**Figure 2:**
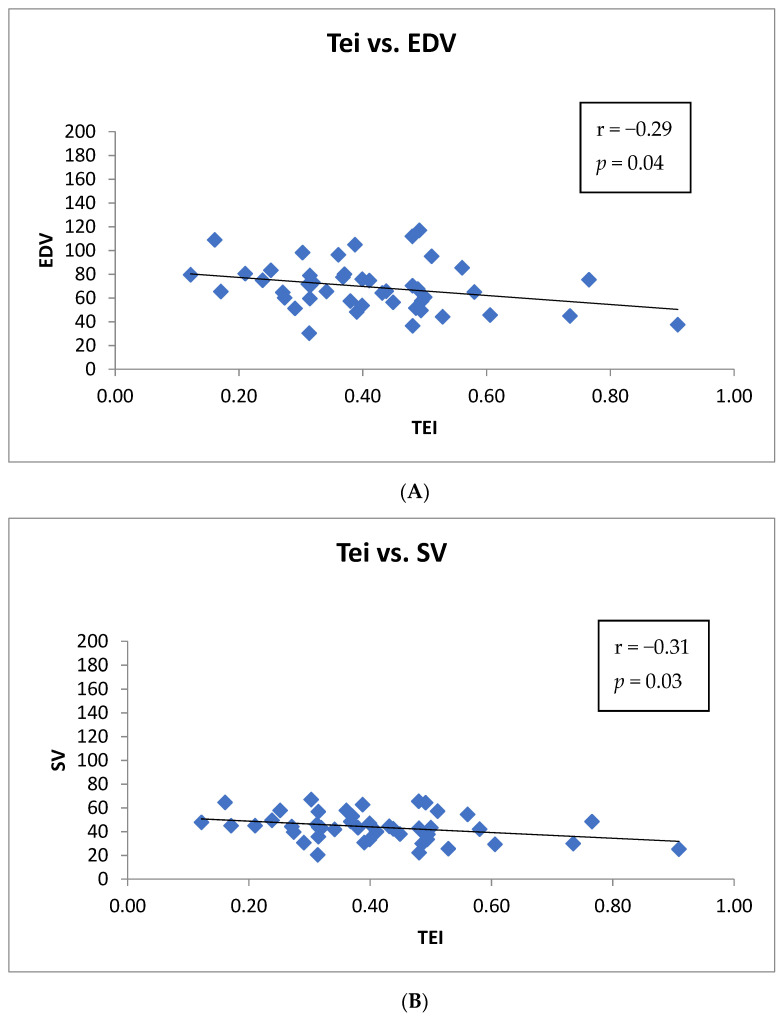
(**A**) Correlation between the Tei index value and the end diastolic volume (EDV) after the first cycle of chemotherapy in all patients (Tei- and Tei+). (**B**) Correlation between Tei index value and stroke volume (SV) after the first cycle of chemotherapy in all patients (Tei- and Tei+).

In all patients, this relationship between Tei index value and EDV and between Tei index value and SV was present after the first cycle of chemotherapy, but with less statistical significance (r = −0.29; *p* = 0.04 and r = −0.31; *p* = 0.03, respectively) (Figure 2, panel A and B). Instead, there was not a significant correlation between the baseline Tei index value and the LVEF value, also measured at 12-months follow-up.

## 4. Discussion

Echocardiography remains the most widely used technique for the evaluation of cardiotoxicity in chemotherapy-treated patients [6]. Our study considered the effectiveness of the Δ-Tei index in early identification of patients with high risk of developing anthracycline-related cardiotoxicity. Left ventricular Tei index is a complex echocardiographic/doppler index of myocardial performance, attesting to the presence of ventricular dysfunction [13].

The population enrolled in the study is homogeneous for characteristics and treatment (ABVD and R-CHOP protocols) for HL and NHL. None of the patients enrolled in the study showed signs or symptoms of heart failure or a significant reduction of LVEF. This data suggest that the studied population had no clinical or echocardiographic signs of established anthracycline-related cardiotoxicity.

No statistically significant correlation was found between Tei index value and LVEF in either of the two groups of patients with low (Tei-) and with high (Tei+) index. This data can be easily explained because the Tei index represents an early marker of ventricular dysfunction, so its increase does not significantly impair the LVEF. This result is in accordance with the study conducted by Senju et al. that found an increase of the Δ-Tei index in 23 doxorubicin-treated patients affected by hematologic neoplasms, irrespective of the LVEF value. The study confirmed the greater sensitivity of the Tei index than the LVEF in identifying early anthracycline-related cardiotoxicity [12].

In the Tei- group, a significant increase of the LV EDV and LV ESV, as well as a consequent increase of stroke volume (SV), were observed. This result may reflect an effective compensatory mechanism to a “cardiac insult” represented by anthracyclines with a dilatation of ventricular chambers and consequent increase of SV in patients without alterations of the LV systolic and diastolic function (Tei-). In such patients, in fact, the administration of anthracycline may represent the trigger for the establishment of compensatory mechanisms with the aim of maintaining unchanged SV, even in the presence of a cardiotoxic drug. This compensation mechanism is in agreement with the results reported by Lipshultzet al., which revealed an increase of ventricular afterload in children affected by ALL subjected to doxorubicin therapy [16].

The increase in EDV and ESV values was not recognized in the group of Tei+ patients. Probably, the latter proved incapable of compensatory mechanisms producing an increase of ventricular volumes. For this incompetence of the compensatory mechanism, we think that the Tei+ patients presented a greater risk of anthracyclines-related cardiotoxicity.

In our study, an inverse correlation between the Tei index and EDV at 12 months and between the Tei index and SV was observed. This inverse correlation suggests that increases of ventricular volumes, in order to maintain an appropriate SV, are a prerogative of patients with a good systolic and diastolic function (Tei-). This significant correlation is already evident just after the first cycle of chemotherapy, though with a lower statistical significance, highlighting that a functional heart (Tei-) is already able to modify its geometry even after the first chemotherapeutic “cardiac damage”.

Based on these findings, the Tei index could represent an effective and valid echocardiographic marker able to identify asymptomatic patients at increased risk of developing anthracyclines-related cardiotoxicity, more sensitively than LVEF.

No statistically significant correlation was found between the value of Tei and the increase of anthracyclin doses. Ishii et al. evaluated the validity of the Tei index in identifying the presence of subclinical cardiotoxicity in a population of 65 anthracycline-treated children. The study showed that the Tei index value tends to increase even for moderate doses of anthracycline (≥200 mg/m^2^), enabling us to identify those patients who are at higher risk of developing cardiotoxicity [17]. In the adult population, Belham revealed an increase of the Tei index even after low doses of anthracycline (doxorubicin 50–125 mg/m^2^), underlining the presence of a “continuum”of anthracycline cardiac damage [18].

Bennet et al., in their systematic review, concluded that there are some studies that suggest that the Tei index may be useful indicator of early cardiotoxicity, but the findings are inconsistent [19]. In a recent work, Naderi et al. demonstrated a change in the Tei index in children after treatment with anthracycline combinations [20].

The main limitation of our study is a relatively small population, even if homogeneously treated. We need further experiences with a larger population to demonstrate whetherthe Tei index value can be used daily in a correct management of cardiotoxicity in lymphoma patients, so as to identify the high-risk patients to whom cardioprotective drugs (i.e., ACEi and BB) should be prescribed to prevent a clinical heart failure. It might be interesting to evaluate the possible use of this measure as an alternative or complementary to LVEF or strain parameters such as global longitudinal LV systolic strain. However, even if in a small but strictly selected population, our study confirms the recognized role of the Tei index forearly identification of asymptomatic patients without significant reduction of LVEF at high risk of developing anthracycline-related cardiotoxicity.

An individual stratification of the risk of cardiotoxicity that includes the Teiindex could help to personalize the therapies and guide the follow-up of patients.

## 5. Conclusions

In conclusion, the Tei index could be a simple, easy to obtain and reliable measurement to monitor cardiac toxicity due to chemotherapy. Owing to its characteristics of easy-acquisition and low-level technical requirement, it may gain popularity amonghigh-volume hospitals.

The Tei index evaluation during a standard 2D-echocardiogram can be used to identify a patient’s group at high risk for cardiotoxicity because it reveals the correlation between the systolic anddiastolic function measured with the Tei index and EDV during anthracycline therapy, as well as the correlation between the Tei index and SV at follow-up. Furthermore, this correlation between the Tei index and SV showed even after the first cycle of therapy, like an early marker of cardiac damage.

## Figures and Tables

**Table 1:** Baseline characteristics.

Variable	n(%) or Median(IQR)
**Sex**	
F	26(63)
M	15(37)
**Age (years)**	50(36–56)
**BSA (m^2^)**	1.73(1.64–1.84)
**Comorbidities**	
**Hypertension**	
No	34(83)
Yes	7(17)
**Smoke**	
No	33(80)
Yes	8(20)
**Diabetes**	
No	40(98)
Yes	1(2)
**Dyslipidemia**	
No	39(95)
Yes	2(5)
**Thyreopathy**	
No	35(85)
Yes	6(15)
**Cardiovascular therapy**	
**ACE inhibitors and/or ARBs**	
No	39(98)
Yes	2(2)
**Diuretics**	
No	37(90)
Yes	4(10)
**Beta-blockers**	
No	40(98)
Yes	1(2)
**Creatinine (mg/dL)**	0.74(0.65–0.8)
**GFR (mL/min/1.73 m^2^)**	108(86–129)
**BNP (pg/mL)**	67(27–90)
**Hystology**	
HL	17(42)
NHL	24(58)
**Grade**	
1	6(15)
2	14(34)
3	12(29)
4	9(22)
**DOX**	
ABVD	17(42)
R-CHOP	24(58)
**Reduced DOX dose**	
No	30(73)
Yes	11(27)
**Cycles**	6
**Cumulative DOX dose (mg/m^2^)**	300(220–300)
**Radiation**	
No	33(80)
Yes	8(20)
**Anthracycline other than DOX**	
No	38(93)
Yes	3(7)

Median values (IQR) or percentage (%); GFR-EPI, glomerular filtration rate by epidemiology collaboration (EPI) formula; BNP, brain natriuretic peptide; HL, Hodgkin’s lymphoma; NHL, non-Hodgkin’s lymphoma; DOX, doxorubicin; ABVD, doxorubicin, bleomycin, vinblastine, dacarbazine; R-CHOP, rituximab, cyclophosphamide, doxorubicin, vincristine, prednisolone.

**Table 2:** Baseline characteristics in Tei- and Tei+ patients.

Variable	Tei	Tei	*p*-Value
	20	21	
**Sex**			
F	13 (65)	13 (62)	0.837
M	7 (35)	8 (38)	
**Age (years)**	50 (29–52.5)	53 (43–64)	0.108
**BSA (m^2^)**	1.78 (1.58–1.91)	1.71 (1.64–1.79)	0.766
***Hypertension***			
No	16 (80)	18 (86)	0.627
Yes	4 (20)	3 (14)	
***Diabetes***			
No	19 (95)	21 (100)	0.299
Yes	1 (5)	0	
No	17 (85)	18 (86)	0.948
Yes	3 (15)	3 (14)	
**Cardiovascular therapy**			
***ACE inhibitors and/or ARBs***			
No	20 (100)	19 (90.5)	0.157
Yes	0	2 (9.5)	
***Diuretics***			
No	18 (90)	19 (90.5)	0.959
Yes	2 (10)	2 (9.5)	
***Beta-blockers***			
No	20 (100)	20 (95.2)	0.323
Yes	0	1 (4.8)	
**Creatinine (mg/dL)**	0.75 (0.65–0.80)	0.73 (0.67–0.81)	0.816
**GFR (mL/min/1.73 m^2^)**	113.79 (101.93–130.80)	95.49 (84.83–126.44)	0.165
**BNP (pg/mL)**	72 (33.61–103.23)	34.14 (27.06–85.38)	0.533
**Cumulative DOX dose (mg/m^2^)**	300 (220–300)	300 (281.25–300)	0.485
**Radiation**			
No	14 (70)	19 (90.5)	0.098
Yes	6 (30)	2 (9.5)	
**Anthracycline other than DOX**			
No	17 (85)	21 (100)	0.065
Yes	3 (15)	0	

GFR-EPI, glomerular filtration rate by EPI formula; BNP, brain natriuretic peptide; DOX, doxorubicin.

**Table 3:** Correlation between left ventricular ejection function (LVEF) during chemotherapy and follow-up and basal Tei index value.

LVEF	Tei	r	*p*-Value
**I cycle**	I cycle	0.03	0.821
**II cycle**	I cycle	0.27	0.069
**IIIcycle**	I cycle	0.11	0.469
**IV cycle**	I cycle	−0.07	0.651
**FU 1 m**	I cycle	0.06	0.751
**FU 3 m**	I cycle	0.03	0.872
**FU 6 m**	I cycle	−0.19	0.306
**FU 12 m**	I cycle	0.06	0.806

**Table 4:** Changes of LVEF (A) and stroke volume (SV) (B) during chemotherapy and follow-up.

**A**	**Tei-**			**Tei+**		
**LVEF** **(%)**	***I cycle***	***IV cycle***	***p-value***	***I cycle***	***IV cycle***	***p-value***
	63.86 ± 4.71	62.98 ± 4.80	0.367	63.14 ± 4.78	64.48 ± 5.49	0.372
	***I cycle***	***FU12m***	***p-value***	***I cycle***	***FU 12m***	***p-value***
	63.86 ± 4.71	61.25 ± 6.14	0.131	63.14 ± 4.78	64.58 ± 5.43	0.195
**B**	**Tei-**			**Tei+**		
**SV** **(mL)**	***I cycle***	***IV cycle***	***p-value***	***I cycle***	***IV cycle***	***p-value***
	63.86 ± 4.71	62.98 ± 4.80	0.367	63.14 ± 4.78	64.48 ± 5.49	0.372
	***I cycle***	***FU12m***	***p-value***	***I cycle***	***FU 12m***	***p-value***
	63.86 ± 4.71	61.25 ± 6.14	0.131	63.14 ± 4.78	64.58 ± 5.43	0.195

## Supplementary Materials

The following are available online at https://www.mdpi.com/article/10.3390/jpm12020290/s1, Table S1: Changes of EDV (A) and EBV (B) during chemotherapy and follow-up.
