# Supplementary material for: Echocardiography Monitoring during Anthracycline Administration in Hodgkin and Non-Hodgkin’s Lymphoma: The Tei Index Evaluation
Source: J Pers Med. 2022 Feb 16;12(2):290. doi: 10.3390/jpm12020290 (PMC8880655; doi:10.3390/jpm12020290)
Supplement: Supplementary file 1 [file jpm-12-00290-s001.zip › jpm-1551163-supplementary.pdf]

Table S1. Changes of EDV (A) and EBV (B) during chemotherapy and follow up.

A	*TEI-*			*TEI+*		
	I cycle	***IV cycle***	***pvalue***	***I cycle***	***IV cycle***	***pvalue***
EDV (ml)	*67.51±24.59*	*73.14±25.73*	*0,04*	*71.1±15.23*	*67.62±14.57*	*0,411*
	I cycle	***FU***	***pvalue***	***I cycle***	***FU 12m***	***pvalue***
	67.51±24.59	*68.25±19.75*	*0,375*	*71.1±15.23*	*72.71±14.84*	*0,109*

B	*TEI-*			*TEI+*		
	I cycle	***IV cycle***	***pvalue***	***I cycle***	***IV cycle***	***pvalue***
ESV (ml)	*25.03±11.71*	*27.42±10.82*	*0,02*	*26.17±6.58*	*24.08±6.75*	*0,111*
	I cycle	***FU***	***pvalue***	***I cycle***	***FU 12m***	***pvalue***
	25.03±11.71	*27.16±11.24*	*0,129*	*26.17±6.58*	*25.55±5.71*	*0,461*
